# Dietary *Bacillus subtilis* Group Reduces the General Infection of *Salmonella* Pullorum in Broiler Chicken

**DOI:** 10.3390/antibiotics15040389

**Published:** 2026-04-10

**Authors:** Yunsheng Chen, Hanqing Li, Xuechun Zhang, Jianfei Zhu, Jijun Kang, Kui Zhu

**Affiliations:** 1 State Key Laboratory of Veterinary Public Health and Safety, College of Veterinary Medicine, China Agricultural University, Beijing 100193, China; 2 Technology Innovation Center for Food Safety Surveillance and Detection (Hainan), Sanya Institute of China Agricultural University, Sanya 572025, China

**Keywords:** *Salmonella* Pullorum, *Bacillus subtilis*, chicken, antibacterial activity, *Bacillus amyloliquefaciens*

## Abstract

**Background:** *Salmonella enterica* subsp. *enterica* serovar Gallinarum biovar Pullorum (*Salmonella* Pullorum) is a specific avian pathogen responsible for Pullorum disease, causing substantial economic losses to the global poultry industry. With the rising restrictions on antibiotic use, probiotics have emerged as promising therapeutic alternatives. The *Bacillus subtilis* group, including *B. amyloliquefaciens* and *B. subtilis*, is a collection of closely related species that has been widely used as a probiotic due to its broad-spectrum antimicrobial activity and other benefits. However, how the probiotics-derived antibacterial phenotype contributes to infection control is still unclear. **Methods:** In this study, we used two different antibacterial phenotype strains, *B. amyloliquefaciens* and *B. subtilis*, to treat *S.* Pullorum infections. The spores of two strains (10^7^ CFUs) were supplemented daily for 21 days. **Results:** The reduction in body weight gains and the severity of *S*. Pullorum-induced symptoms were ameliorated. Compared to *B. subtilis*, *B. amyloliquefaciens* exhibited a stronger host protection effect, manifested in a greater reduction in the bacterial load of *S*. Pullorum in organs throughout the infection. Furthermore, both strains enhanced cecal microbiota diversity, suppressed infection-associated taxa, and promoted beneficial genera. **Conclusions:** Our findings demonstrate that probiotic *Bacillus* can alleviate *S*. Pullorum infection and improve growth performance in poultry, especially the antimicrobial phenotype contributing to pathogen clearance. This work provides crucial insights for developing effective, probiotic-based strategies against Pullorum disease.

## 1. Introduction

*Salmonella* Pullorum is a major avian pathogen responsible for general and septicemic infections, clinically manifested as Pullorum disease [1]. The disease is characterized by high mortality in chicks, reduced egg production in adult hens, and reproductive system damage, thereby causing substantial economic losses to the poultry industry [2,3]. While antibiotics such as enrofloxacin and amoxicillin are standard control measures, their extensive use in intensive farming has accelerated the emergence of multidrug resistance. Notably, resistance rates to nalidixic acid have reached 90% [4,5], underscoring the urgent need for effective, non-antibiotic therapeutic alternatives.

Although strategies including bacteriophage therapy, antimicrobial peptides, and phytogenics have been explored, their application is frequently constrained by instability, low efficiency, or prohibitive costs [6]. In contrast, probiotics, particularly spore-forming *Bacillus* species, exhibit advantages including environmental stability under stress conditions, compatibility with oral administration, and multifaceted mechanisms of action, establishing them as compelling candidates for sustainable therapeutic interventions in infection prevention [7]. *Bacillus subtilis* (i.e., *B. subtilis* group) has been extensively applied as a probiotic feed additive owing to its long history of high safety and various beneficial properties, including antimicrobial activity [8,9], host modulation [10,11], and colonization resistance [9,12]. The *B. subtilis* group encompasses multiple species such as *B. subtilis*, *B. amyloliquefaciens*, *B. velezensis*, and *B. licheniformis*. These species are close in phylogenetic distance and share certain probiotic characteristics. Nevertheless, the extent of these beneficial effects is strain-dependent, largely influenced by specific physiological traits and the type and abundance of secreted metabolites. Among the various probiotic functions of *B. subtilis*, its antimicrobial phenotype has attracted particular attention. Distinct antimicrobial metabolites provide *B. subtilis* with competitive advantages within microbial communities, such as polyenes that disrupt biofilms [13], lipopeptides that inhibit quorum sensing [9], and cyclic peptides that chelate iron [14]. Moreover, a few studies have shown *B. subtilis* group strains are effective in treating *Salmonella* infection by maintaining the expression of tight junction proteins and mucin protein and balancing cytokine levels [15,16]. However, most studies have focused primarily on the in vitro antimicrobial activities of *B. subtilis*, and how antimicrobial substances or phenotype contribute to host health is still largely unexplored.

The present study addresses this gap by investigating the effects of different antibacterial phenotypes of *B. subtilis* on the treatment of *S*. Pullorum infections in chickens. Through in vitro and in vivo experiments, we intend to systematically evaluate how strains with distinct antimicrobial profiles influence the progression of Pullorum disease. Our findings elucidate the role of antibacterial phenotypes in infection control, providing a scientific basis for the rational selection of *B. subtilis* strains for poultry health management, and provide an alternative strategy to prevent and treat Pullorum disease in a probiotic-based way.

## 2. Results

### 2.1. Antibacterial Potential of B. subtilis

**Figure 1:**
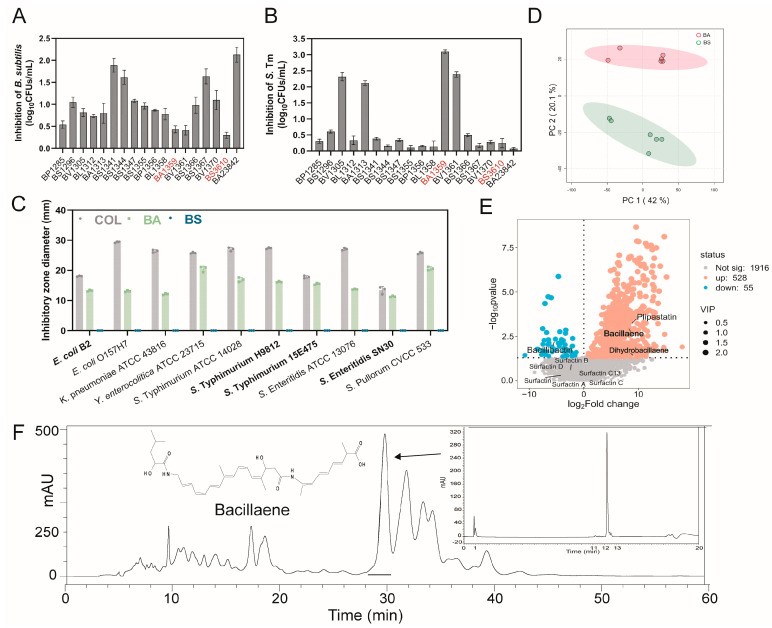
Screening and metabolic characterization of antimicrobial *Bacillus strains*. (**A**) Growth inhibition of *B. subtilis* group strains by *S*. Typhimurium in co-culture. Values represent the reduction in *Bacillus* cell counts (log_10_ CFU/mL). (**B**) Inhibitory effect of various *B. subtilis* strains against *S*. Typhimurium ATCC 14028, quantified by the decrease in pathogen cell counts. (**C**) Antimicrobial activity of secondary metabolites (5 mg) extracted from BA and BS against a panel of pathogenic and multidrug-resistant (MDR) bacteria. Colistin (10 µg) served as the positive control. MDR strains are highlighted in bold. (**D**) Principal component analysis of metabolite samples from two strains. Each ellipse was drawn at a 95% confidence level to include most samples in either group. (**E**) Volcano plot visualizing differentially abundant metabolites between BA and BS. Key antimicrobial compounds with significant variable importance in projection (VIP > 1) are labeled. (**F**) Identification of the active antimicrobial agent. The bioactive fraction from BA was purified via HPLC and identified as bacillaene. The inset displays the chromatogram verifying the purity of the isolated peak. Data are presented as mean ± s.d. derived from three biologically independent experiments (*n* = 3).

To identify the most effective *B. subtilis* strain capable of antagonizing *Salmonella* species growth, we utilized eighteen *B. subtilis* strains from our probiotic library [17]. Co-cultivation assays indicated that *S*. Typhimurium generally inhibited the growth of *B. subtilis* strains, with colony counts decreasing by 0.30 to 2.13 log_10_ CFUs/mL. However, a few isolates displayed relative resilience to this pathogen-induced suppression; these included *B. amyloliquefaciens* (strain BA1359, hereafter BA) and *B. subtilis* (strain NCIB3610, hereafter BS), which were relatively less inhibited by *S.* Typhimurium (Figure 1A). In contrast, the inhibitory capability of *Bacillus* strains against *S*. Typhimurium was highly variable. While most isolates exhibited limited activity, a specific subset strongly suppressed pathogen growth by 2.11 to 3.10 log_10_ CFUs/mL (Figure 1B). Based on these distinct reciprocal interaction patterns—where BA served as a robust antagonist and BS as a resilient non-antagonist—we selected these two strains for subsequent analyses.

To investigate the key metabolite underlying the antimicrobial phenotype differences between BA and BS, we extracted secondary metabolites from both strains and assessed their inhibitory activity against a panel of 10 drug-resistant pathogenic Enterobacteriaceae. The organic extracts from BA exhibited broad-spectrum antimicrobial activity against multiple pathogens, whereas BS extracts showed no inhibitory effect (Figure 1C). To screen the most differential metabolite responsible for the inhibitory effect, we performed comparative metabolomics on both strains. Principal component analysis (PCA) confirmed a significant divergence in the global metabolite landscapes of BA and BS (Figure 1D). Further differential analysis identified 1916 metabolites that were significantly enriched in BA relative to BS. Notably, the volcano plot highlighted the upregulation of known antimicrobial compounds, including plipastatin and bacillaene (along with its derivative, dihydrobacillaene) (Figure 1E). To pinpoint the active constituent, we performed bioactivity-guided fractionation using High-Performance Liquid Chromatography (HPLC) and mass spectrometry, which identified bacillaene as the primary antimicrobial agent (Figure 1F and Figure S2, Table S2). Consistent with previous reports on the efficacy of polyenes against Gram-negative bacteria [13], these data suggest that the superior in vitro antibacterial activity of BA is driven by the elevated production of bacillaene.

### 2.2. Administration of Bacillus Spores Ameliorated the Weight Gain Loss Challenged by S. Pullorum

**Figure 2:**
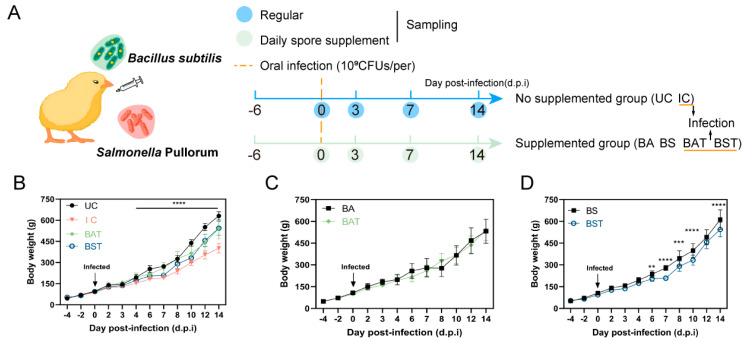
Protective efficacy of *Bacillus* strains against *S*. Pullorum-induced weight loss in broilers (**A**) Schematic representation of the experimental design for *S*. Pullorum infection and dietary *B. subtilis* supplementation. (**B**–**D**) Body weight trajectories of broiler chickens. (**B**) Comparison of body weights among UC, IC, and infected groups treated with BA (BAT) or BS (BST). (**C**,**D**) Pairwise comparison of weight gain between infected (BAT/BST) and their respective uninfected, spore-supplemented counterparts. Data were shown as mean ± SD, *n* ≥ 6. Student’s *t*-test was performed to compare the means of body weight between groups in same time point, where statistically significant differences were determined (** *p* < 0.01, *** *p* < 0.001, **** *p* < 0.0001). For panel (**B**), statistically significant differences were performed between groups by one-way ANOVA with Tukey’s post hoc tests).

Based on the in vitro antimicrobial activity, we assessed their protective effects against *S*. Pullorum infection in a 21-day broiler chicken trial. Spores were administered ad libitum via drinking water (10^7^ CFU/mL) (Figure 2A). Following infection, the Infection Control (IC) group exhibited significant growth retardation compared to the Uninfected Control (UC) group starting from day 4 (*p* < 0.0001). While spore administration did not fully restore growth to baseline UC levels, both the BA-treated (BAT) and BS-treated (BST) groups significantly outperformed the IC group by day 8 post-infection (Figure 2B). To dissect this variance, we benchmarked the growth of treated cohorts against their respective uninfected, spore-supplemented counterparts. The BAT group demonstrated better protection, maintaining weight gain comparable to the uninfected BA group throughout the trial (Figure 2C). In contrast, the BST group conferred only partial protection; while it improved outcomes relative to IC, it failed to fully restore growth to baseline levels, exhibiting a significant weight deficit compared to the uninfected BS group starting from day 6 (Figure 2D). These findings highlight the superior therapeutic efficacy of BA over BS.

### 2.3. BS and BA Effectively Decreased the Abundance and Infection of S. Pullorum

**Figure 3:**
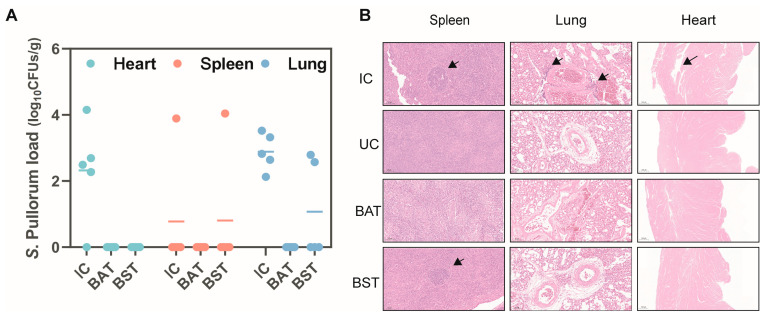
Mitigation of systemic *S*. Pullorum colonization and tissue pathology by *Bacillus* strains. (**A**) Quantification of *S*. Pullorum bacterial burden in the heart, spleen, and lungs at 14 dpi. Data represent mean values, *n* = 5. (**B**) Representative hematoxylin and eosin (HE)-stained histopathological sections of the indicated organs. Black arrows denote inflammatory cell infiltration and focal necrotic lesions. Scale bars: 500 µm (2× magnification) and 50 µm (20× magnification).

Systemic dissemination and multi-organ colonization are hallmarks of *S*. Pullorum infection, leading to extensive tissue damage [18]. To evaluate therapeutic efficacy, we quantified bacterial burden and assessed histopathological changes in target organs (heart, spleen, and lungs) at 14 days post-infection (dpi). In the IC group, *S*. Pullorum established robust colonization, particularly in the heart and lungs (Figure 3A). This high bacterial burden was associated with severe pathology, including splenic granulomas and pulmonary congestion characterized by inflammatory cell infiltration. Notably, the heart exhibited gross white necrotic nodules, which corresponded to pronounced myocardial disintegration and necrosis in histological sections (Figure 3B and Figure S3). Dietary spore supplementation significantly mitigated this systemic invasion. Most strikingly, the *S.* Pullorum was undetectable in the sampled tissues of the BAT group, such as heart, spleen, and lungs, thereby preventing the associated histopathological lesions.

**Figure 4:**
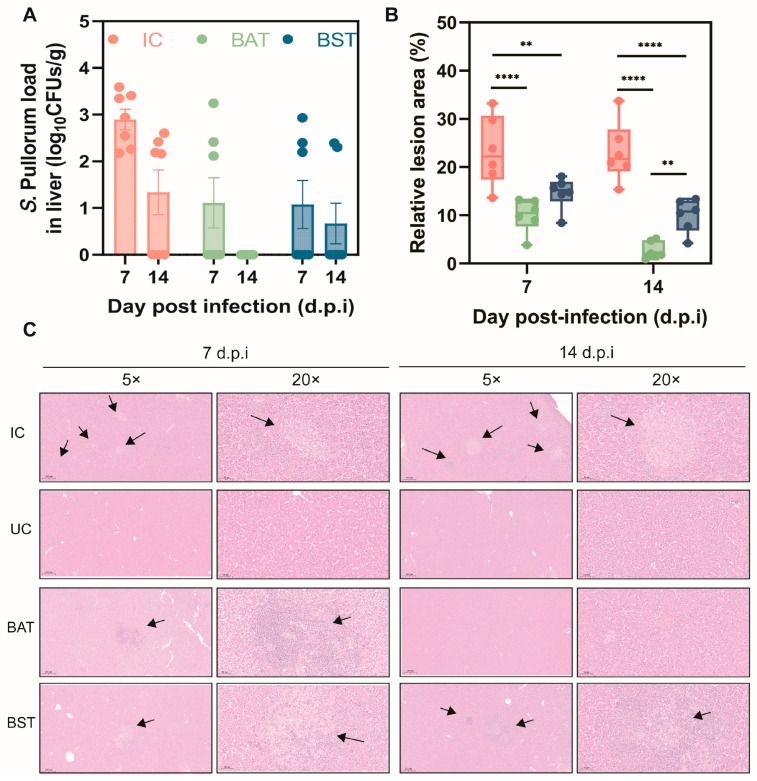
Attenuation of hepatic colonization and tissue necrosis by *Bacillus* treatment. (**A**) Quantification of *S*. Pullorum bacterial burden in the liver at 3, 7, and 14 dpi. (**B**) Quantification of relative necrotic lesion area in the liver at 7 and 14 dpi. (**C**) Representative HE-stained liver sections showing tissue pathology. Black arrows indicate focal necrotic lesions and inflammatory infiltration. Scale bars: 200 µm (left panels) and 50 µm (right panels). Data present mean ± s.d (*n* ≥ 6). Statistical significance was determined by two-tailed Student’s *t*-test (** *p* < 0.01, **** *p* < 0.0001).

Given the liver’s susceptibility to *S*. Pullorum, we monitored hepatic colonization and pathology dynamics at 3, 7, and 14 days dpi (Figure 4A). At 3 dpi, no bacteria were detected in the liver, likely due to a lag in early colonization. By 7 dpi, however, infection in the IC group worsened, characterized by extensive white necrotic areas on the liver surface (Figure S3) and a mean bacterial load of 2.89 ± 0.58 log_10_ CFUs/g. Histological analysis showed severe cell lysis and loss of nuclei in these regions, with necrosis covering up to 33.21% of the liver area (Figure 4C). At 14 dpi, despite bacteria being naturally cleared in some IC birds, the extent of liver necrosis remained significantly higher compared to the spore-treated groups (BAT and BST) (*p* < 0.001, Figure 4B). In contrast, the BAT group showed a significant reduction in both bacterial load and tissue damage at 7 and 14 dpi. Notably, signs of recovery—including cell regeneration and intact nuclei—were evident in the BAT group, mirroring the improvements seen in the BST group by day 14 (Figure 4C). Collectively, supplementation with either BA or BS spores effectively mitigated hepatic colonization and limited infection-induced tissue damage.

### 2.4. Bacillus Dietary Supplementation Restores Gut Microbiota

**Figure 5:**
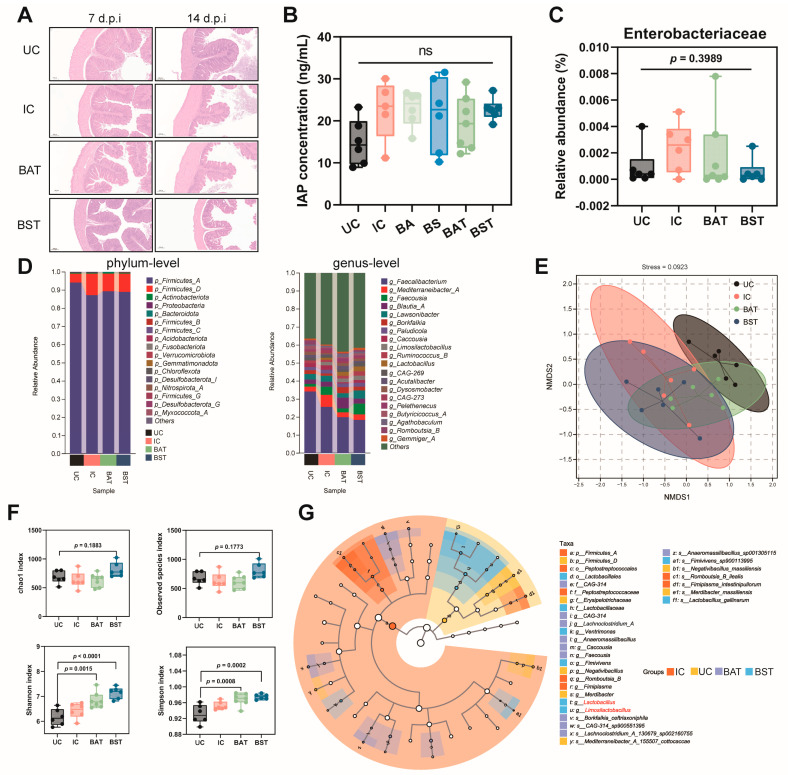
Restoration of intestinal homeostasis and microbiota diversity by *Bacillus* supplementation. (**A**) HE images of cecum section from the corresponding treatment, Scale bars: 200 µm. (**B**) Quantification of serum intestinal alkaline phosphatase (IAP) levels, *n* ≥ 5. (**C**) Relative abundance of the family Enterobacteriaceae in cecal microbiota, *n* ≥ 6. (**D**) Taxonomic profiles showing the relative abundance of the top 20 bacterial taxa at the phylum and genus levels. (**E**) Non-metric multidimensional scaling (NMDS) plot of cecal microbiota based on binary Jaccard distances. (**F**) Alpha diversity analysis (*n* ≥ 6). Chao1 and Observed species indices reflect species richness, while Shannon and Simpson indices reflect microbial diversity. (**G**) LEfSe cladogram identifying differentially abundant taxa. Node size corresponds to relative abundance; colored nodes indicate taxa significantly enriched in the specific group, while hollow nodes indicate no significant difference. The probiotic species are indicated in red front. LDA threshold value ≥ 2. Data present mean ± s.d. Statistical significance was determined by one-way ANOVA with Tukey’s post hoc test.

*Salmonella* infection has previously been reported to compromise the intestinal barrier and disrupt the gut microbiota in broiler chicks [19,20]. To understand the detrimental effects of *S*. Pullorum on the intestine, we examined changes in intestinal morphology on days 7 and 14 post-infection. Unexpectedly, *S*. Pullorum infection caused no visible damage to the intestine; the structure of intestinal villi and the integrity of the intestinal wall remained consistent across all groups (Figure 5A). Consistent with this, the level of intestinal alkaline phosphatase (IAP), a marker of intestinal permeability, revealed no significant differences among the groups (Figure 5B). We therefore hypothesized that the intestinal colonization of *S*. Pullorum might be weak. To test this, we conducted 16S rRNA sequencing to quantify its abundance. The results confirmed that there were no significant differences in the abundance of Enterobacteriaceae (the family encompassing *S*. Pullorum) in infected groups compared to the control group (*p* = 0.3989). Furthermore, the proportion of Enterobacteriaceae accounted for less than 0.01% in the overall microbiota, indicating extremely low-level colonization by *S*. Pullorum (Figure 5C).

Further analysis of the gut microbiota composition revealed that at the phylum level, the microbiota was predominantly composed of Firmicutes, which accounted for over 98% of the total microbial population. *S*. Pullorum infection increased the abundance of Firmicutes (specifically Firmicutes_D), whereas subsequent spore supplementation (both in BA and BS groups) reversed this increase. At the genus level, *S*. Pullorum infection markedly elevated the proportions of Mediterraneibacter and Romboutsia (*p* < 0.05). Notably, spore supplementation (in both BA and BS groups) effectively reduced their abundance, with Mediterraneibacter returning to a level comparable to the UC group (UC vs. BAT: *p* = 0.8432, UC vs. BST: *p* = 0.8055) (Figure 5D). Beta diversity analysis indicated that *S*. Pullorum infection significantly altered the microbiota community structure. Although spore supplementation changed the composition of microbiota, it was unable to fully restore the microbiota to its original state. Regarding alpha diversity, no significant differences were observed in the Chao1 and observed species indices (*p* = 0.1883 and *p* = 0.1773), implying comparable microbial richness across groups. However, a significant increase in the Shannon and Simpson indices following BA and BS interventions indicated that these treatments enhanced microbial diversity (Figure 5F).

To identify specific bacterial taxa driving these community shifts, we performed Linear Discriminant Analysis Effect Size (LEfSe) (Figure 5G). The results showed that *S*. Pullorum infection enriched genera such as *Negativibacillus*, *Lachnoclostridium*, and *Merdibacter*. Notably, spore supplementation markedly suppressed the abundance of these infection-associated genera while concurrently promoting the proliferation of beneficial genera, including *Lactobacillus* and *Limosilactobacillus* (Figure S4). Collectively, these findings indicate that dietary intake of either BA or BS spores not only improves overall microbiota diversity but also enriches the population of beneficial bacteria.

Finally, we evaluated intestinal colonization to understand the difference in protective efficacy between the two strains. While *Bacillus* counts in the cecum generally declined over time, BA maintained a significantly higher and more stable abundance than BS across all intestinal segments. Strikingly, the cecal abundance of BA was 6 log_10_ CFU/g higher than that of BS (Figure S5). This suggests that the superior protective effect of BA against *S*. Pullorum is driven, at least in part, by its robust capacity to colonize and persist in the intestine.

## 3. Discussion

*S*. Pullorum infection remains a persistent challenge in the poultry industry, characterized by its ability to cause systemic dissemination and establish chronic carriage in hosts [21,22]. While antibiotics are currently the primary control method, their long-term use frequently disrupts gut microbiota and increases the risk of coinfection. Consequently, there is an urgent need for non-antibiotic alternatives to restore colonization resistance [23]. Although *B. subtilis* spores have been shown to ameliorate intestinal damage caused by other Salmonella serovars [24,25,26], their specific efficacy and protective mechanisms against *S*. Pullorum remain poorly understood. In this study, we used *S*. Pullorum strain CVCC533 to establish a chicken infection model. Unlike previous reports of high mortality [23,27], we observed no deaths in our chicks, though severe lesions were found in the intestine and liver. This difference might be due to the genetic background or immune status of the chickens. Consistent with previous studies [19], *S*. Pullorum spread to multiple organs (liver, heart, lungs) with high bacterial loads. Interestingly, we found no bacterial shedding in feces. Our 16S rRNA data supported this, showing extremely low *S*. Pullorum abundance (0.01%) in the gut lumen. This suggests that the disease is driven by internal organ invasion rather than persistent gut colonization.

Despite exhibiting distinct in vitro antibacterial phenotypes, both *B. subtilis* strains conferred robust in vivo protection against *S*. Pullorum, pointing to a conserved therapeutic mechanism. While the competitive exclusion of enteropathogens is widely attributed to the diverse antimicrobial peptides (AMPs) and bacteriocins secreted by *Bacillus* [28], definitively proving direct metabolite-mediated killing in vivo remains further validation and is sometimes limited by genomic editability and differential expression of metabolites in vivo. Recognizing that survival in the gastrointestinal tract depends on more than microbial interference, we wondered whether certain metabolites participate in host interactions to promote health. Previous studies have reported that the analogs of 2-hydroxy-4-methylpentanoic acid [29] can enhance therapeutic efficacy by reinforcing intestinal barrier integrity, operating in tandem with direct antimicrobial competition. In our study, metabolomic profiling revealed that both strains broadly synthesize 3-methylpentanoic acid, suggesting that the similar protection effects may be attributed to these organic acids.

Consistent with previous reports [24], *B. subtilis* treatment significantly modulated the gut microbiota. Both strains reversed the infection-induced changes and suppressed genera like *Negativibacillus* and *Merdibacter* while promoting beneficial genera such as *Lactobacillus*. However, the abundance of *Lachnoclostridium*, a genus associated with inflammation [30,31], increased in the BAT group. This implies that the strain with strong antibacterial activity might have minor side effects. Nonetheless, the increase in *Lactobacillus* and overall microbial diversity (Shannon and Simpson indices) indicates that *B. subtilis* partially restores intestinal homeostasis and colonization resistance against pathogens [32]. Crucially, this restored colonization resistance is likely driven by synergistic cooperation between the administered *Bacillus* and the endogenous microbiota. The significant enrichment of beneficial taxa like *Lactobacillus* and *Limosilactobacillus* suggests a cooperative dynamic where *B. subtilis* shapes a favorable intestinal niche for these bacteria. In turn, these expanded populations work synergistically with the spores to competitively exclude opportunists and reinforce the intestinal barrier, establishing a resilient microbial network that collectively suppresses S. Pullorum invasion.

We showed that *B. subtilis* spores confer protection against *S*. Pullorum infection through a dual mechanism involving metabolite-mediated barrier enhancement and microbiota restoration. Although our data implicate antimicrobial activity in pathogen elimination, further investigation is required to elucidate how specific metabolites, such as bacillaene, function in vivo and whether the primary protection effect is attributable to bacillaene, which may exert both antibacterial and immunomodulatory effects. Overall, this work validates the utility of *B. subtilis* spores as a viable antibiotic alternative for poultry health management.

## 4. Materials and Methods

### 4.1. Bacterial Strains and Culture Conditions

*B. subtilis* strains were derived from probiotic products from diverse origins, except for BA23842 and *B. subtilis* NCIB3610. Indicator strains such as *Escherichia coli*, *Klebsiella pneumoniae*, *Yersinia enterocolitica*, and *Salmonella* species were stocked in our laboratory (Supplementary Table S1). Strains were inoculated into 2 mL of fresh LB broth and incubated overnight at 37 °C with shaking at 200 rpm before the experiment.

### 4.2. Pairwise Competition Assay

*B. subtilis* strains and *S.* Typhimurium were co-inoculated into LB broth in 24-cell plates at a 1:1 initial ratio (2 × 10^6^ CFUs/mL each) and incubated statically at 42 °C for 24 h as previously described [33]. Populations were enumerated on selective agar supplemented with 10 μg/mL colistin (for *B. subtilis*) and 10 μg/mL vancomycin (for *S.* Typhimurium) after incubation at 37 °C for 18 h. Growth inhibition was calculated as the difference in CFU counts between monoculture controls and co-cultures for each biological replicate (*n* = 3).

### 4.3. Extraction and Assessment of Antibacterial Activity

To obtain crude antibacterial extracts, *B. subtilis* NCIB3610 and *B. amyloliquefaciens* CAU1359 were cultured in modified M9 broth for 48 h at 30 °C, 200 rpm in the dark. Cell-free supernatants were extracted three times with ethyl acetate, dried under reduced pressure, and resuspended in 70% methanol. The antibacterial activity was assessed via the Oxford cups diffusion method. Briefly, indicator strains (2 × 10^6^ CFUs/mL) were embedded in the upper nutrient agar layer. Oxford cups were loaded with 50 μL of crude extract, allowed to diffuse at 4 °C for 8 h, and incubated at 37 °C for 18 h. Inhibition zones were measured using a vernier caliper; zones of 10.5–25.0 mm were classified as active.

### 4.4. Isolation and Purification of Antibacterial Components

Crude extracts were fractionated via sequential partitioning with cyclohexane and methanol (20–80%). Bioactive fractions were pooled, dried, and resuspended in 70% methanol. Semi-preparative HPLC was performed on a Shim-pack GIST C18 column (10 × 250 mm, 5 μm, Shimadzu, Kyoto, Japan) using an isocratic elution of 48% acetonitrile (containing 0.1% formic acid) at 3 mL/min for 50 min. Active peaks were further purified using an XBridge^®^ BEH Shield RP18 column (2.1 × 100 mm, 2.5 μm, Waters, Milford, MA, USA) with a methanol/water gradient containing 0.1% formic acid (0.4 mL/min for 15 min).

### 4.5. Untargeted Metabolomics

Metabolite extraction: *Bacillus* strains were cultured in LB broth (20 mL, 30 °C, 200 rpm, 24 h). Cell-free supernatants were collected, acidified to pH 2 with HCl, and precipitated overnight at 4 °C. Precipitates were harvested, resuspended in methanol (1 mL), and ultrasonicated (30 min, ice bath) with intermittent vortexing. Extracts were filtered (0.22 μm) before analysis [34].

LC-MS analysis: Chromatographic separation was performed on an ACQUITY UPLC HSS T3 column (Waters, Milford, MA, USA) at a flow rate of 0.3 mL/min. Mobile phases consisted of water (A) and acetonitrile (B), both containing 0.1% formic acid. The gradient elution was: 0–2 min, 1% B; 2–12 min, 1–99% B; 12–18 min, 99% B; 18–20 min, 1% B. Mass spectrometry was conducted in positive ion mode (TOF-MS: 80–2000 Da; MS/MS: 60–2000 Da). Source parameters were: ion source gas 1/2, 50 psi; curtain gas, 30 psi; source temperature, 550 °C; spray voltage, 5500 V; declustering potential (DP), 80 V; and collision energy (CE), 20, 35, and 50 V.

Data processing: Raw data were processed using MS-DIAL (v5.5.241113) against MSMS-Public and GNPS databases. Parameters included: minimum peak height > 3000, MS/MS abundance threshold > 100, and sample coverage ≥ 20%. Metabolite intensities were normalized to total ion current (TIC).

### 4.6. Spore Preparation

*Bacillus* strains were inoculated (1%) into DSM medium and cultured at 37 °C (200 rpm) for 48 h to induce sporulation. Vegetative cells were inactivated by heat treatment (80 °C, 30 min). Spores were harvested (10,000× *g*, 30 min, 16 °C), washed three times with PBS, and resuspended in 10% glycerol to 1/10 of the original volume. Spore concentrations were adjusted to 1 × 10^7^ CFUs/mL for animal experiments.

### 4.7. Ethics Approval

All experimental work was approved by the Animal Ethics Committee at China Agricultural University under approval number AW61705202-2-07 in accordance with the guidelines specified in GB/T 35892-2018 [35].

### 4.8. Animal Experiment Design

One-day-old *Salmonella*-free broiler chicks (35 ± 4.70 g) were obtained from a commercial hatchery and randomly allocated into six groups (*n* = 18–24 per group) following established protocols [30,36]. The experiment was conducted in the animal facility of the Veterinary Teaching Hospital, China Agricultural University. As the chicks were acquired at one day of age, the initial 5 days before the bacterial challenge served as the acclimatization period. During the entire experimental period, birds were maintained under standard facility conditions and had ad libitum access to feed (Purina non-medicated starter feed) and water (regular sterile water). To minimize environmental confounders and prevent cross infection, each group was randomly housed in a separate isolator to avoid transmission of the pathogen or spores. The experimental groups were defined as follows: an uninfected control (UC, *n* = 18), an infected control (IC, *n* = 24), two uninfected spore-treated groups (BS and BA, *n* = 18), and two infected spore-treated groups (BST and BAT, *n* = 24). From day 1, the treated groups (BS, BA, BST, and BAT) received drinking water supplemented with *Bacillus* spore (1 ×10^7^ CFUs/mL). At 6 days of age, birds in the IC, BST, and BAT groups were orally challenged with 1 × 10^9^ CFU of *S*. Pullorum, while the UC, BS, and BA groups remained unchallenged.

### 4.9. Physiological Monitoring and Bacterial Load Enumeration

Body weight, feed intake, and water intake were recorded every two days. At 3, 7, and 14 days post-infection, five birds per group were euthanized for tissue collection (duodenum, jejunum, ileum, cecum, liver, spleen, heart, and lungs). Tissue samples were weighed, homogenized in PBS, and serially diluted for bacterial enumeration. *Bacillus* were enumerated on polymyxin-containing agar (37 °C, 24 h), while *S*. Pullorum was counted on Chromagar^®^ *Salmonella* plates (Paris, France) at 42 °C for 24 h. For histopathology, tissues were stained with hematoxylin and eosin (HE). Lesion areas were quantified using Fiji software v1.54p by calculating the ratio of white lesion area to total section area.

### 4.10. Cecal Microbiota Analysis

Cecal contents were collected at 14 d.p.i., flash-frozen, and stored for DNA extraction using the E.Z.N.A DNA Kit (Omega Bio-tek, Norcross, GA, USA). The V3–V4 region of the 16S rRNA gene was amplified (primers 338F/806R), purified, and sequenced on the Illumina MiSeq platform (San Diego, CA, USA). Microbial community analysis was performed using QIIME2 [37] and R (v4.3.3) [38]. Alpha diversity (Chao1, Observed species, Shannon, Simpson, Faith’s PD, Pielou’s evenness, Good’s coverage) was calculated and visualized via box plots and ranked abundance curves [39]. Beta diversity was assessed using Jaccard, Bray–Curtis, and UniFrac distances, visualized through PCoA, NMDS, UPGMA clustering, and PCA. Group differences were tested with PERMANOVA, ANOSIM, and Permdisp. Taxonomic composition was visualized with MEGAN and GraPhlAn. Shared/unique ASVs were displayed in Venn diagrams. Differential abundance was analyzed using MetagenomeSeq v3.11 (Manhattan plots), LEfSe (v1.1.2), and OPLS-DA (“muma” R package v1.4). Random forest classification with 5-fold cross-validation was applied for group discrimination. Co-occurrence networks were inferred via SparCC (pseudocount = 10^−6^), with correlations thresholded using random matrix theory (R package RMThreshold v1.1) and visualized with igraph (R package v2.2.3)/ggraph (R package v2.2.2). Functional potential was predicted with PICRUSt2 against MetaCyc and KEGG databases.

### 4.11. Statistical Analysis

Obtained data were assessed using unpaired Student’s *t* test and one-way ANOVA for 2 or more groups by GraphPad Prism 9 (San Diego, CA, USA). Data were presented as means ± standard deviations. Results were considered significant if the *p* value was less than 0.05.

## Data Availability

The original contributions presented in this study are included in the article and Supplementary Materials. Further inquiries can be directed to the corresponding authors.

## Supplementary Materials

The following supporting information can be downloaded at: https://www.mdpi.com/article/10.3390/antibiotics15040389/s1, Figure S1: The inhibitory effect of S. Pullorum on the growth of both BA and BS was not significant difference across different initial cell numbers; Figure S2: The comparison of purified antibacterial metabolite and bacillaene in MS/MS spectrometry; Figure S3: *S*. Pullorum infection led to the appearance of liver white spots and heart bulges; Figure S4: The relative abundance of *Lactobacillus* and *Limosilactobacillus* across different group; Figure S5: The temporal and spatial distributions of BA and BS in intestinal; Table S1: Bacterial strains used in this study; Table S2. The potential for producing secondary metabolites annotated by antiSMASH. Table S3: The information of top ten largest peak areas identified by MS spectrometry. References [17,19,20,40,41,42] are cited in the Supplementary Materials.
