# Supplementary material for: Dietary Bacillus subtilis Group Reduces the General Infection of Salmonella Pullorum in Broiler Chicken
Source: Antibiotics (Basel). 2026 Apr 10;15(4):389. doi: 10.3390/antibiotics15040389 (PMC13113059; doi:10.3390/antibiotics15040389)
Supplement: Supplementary file 1 [file antibiotics-15-00389-s001.zip › antibiotics-4208634-supplementary.pdf]

Dietary *Bacillus subtilis* group reduces the general infection of *Salmonella* Pullorum in broiler chicken

Yunsheng Chen[1], Hanqing Li[1,2], Xuechun Zhang[1], Jianfei Zhu[1], Jijun Kang[1,2*], Kui Zhu[1,2*]
[1] State Key Laboratory of Veterinary Public Health and Safety, College of Veterinary Medicine, China
[2] Technology Innovation Center for Food Safety Surveillance and Detection, Hainan, Sanya Institute of China Agricultural University. Sanya 572025, China
[*] Corresponding author: Jijun Kang (kangjijun@cau.edu.cn); Prof. Kui Zhu (zhuk@cau.edu.cn), ORCID: Kui Zhu, 0000-0001-8242-3952

Supporting Information

Supplementary Tables (1- 3)
Supplementary Figures (1-5)
Supplementary Methods
Supplementary References

Table S1. Bacterial strains used in this study

**Species**	**Strain designation**	**Name in short**	**Source**	**Reference**
B. paralicheniformis	CAU1285	BP1285	Probiotic product	[40]
B. subtilis	CAU1296	BS1296		
B. velezensis	CAU1305	BV1305		
B. licheniformis	CAU1312	BL1312		
B. amyloliquefaciens	CAU1313	BA1313		
B. subtilis	CAU1341	BS1341		
B. subtilis	CAU1344	BS1344		
B. subtilis	CAU1347	BS1347		
B. subtilis	CAU1355	BS1355		
B. paralicheniformis	CAU1356	BP1356		
B. licheniformis	CAU1358	BL1358		
B. amyloliquefaciens	CAU1359	BA1359		
B. velezensis	CAU1361	BV1361		
B. subtilis	CAU1366	BS1366		
B. subtilis	CAU1367	BS1367		
B. velezensis	CAU1370	BV1370		
B. subtilis	NCIB3610	BS3610	Purchased from China Center of Industrial Culture Collection	[41]
B. amyloliquefaciens	ATCC 23842	BA23842		[42]
E. coli	ATCC 25922		Laboratory stocked	[43]
E. coli	B2			
K. pneumoniae	ATCC 43816			
Y. enterocolitica	ATCC 23715			
S. Typhimurium	ATCC 14028			
S. Typhimurium	H9812			
S. Typhimurium	15E475			[44]
S. enteritidis	ATCC 13076			
S. enteritidis	SN30			
S. Pullorum	CVCC 533			[45]

Table S2. The potential for producing secondary metabolites annotated by antiSMASH.

	B. subtilis NCIB3610		*B. amyloliquefaciens* CAU1359	
Most similar known cluster	Similarity score	Matched region type and cluster length (bp)	Similarity score	Matched region type and cluster length (bp)
surfactin	0.71	NRPS, 65,392	0.82	NRPS, 65,408
plantazolicin	/	/	0.87	RiPP, 23,178
macrolactin	/	/	0.88	PKS, 88,213
bacillaene	0.77	NRPS-PKS, 114,759	0.90	NRPS-PKS, 110,121
plipastatin	0.87	NRPS, 73,389	0.81	NRPS, 77,062
difficidin	/	/	0.92	PKS, 106,167
bacillomycin D	/	/	0.88	NRPS-PKS, 60,768
bacillibactin	0.98	NRPS, 66,289	0.94	NRPS, 65,414
bacilysin	0.84	Other, 41,419	0.98	Other, 41,419
pulcherriminic acid	1.00	CDPS, 20,747	/	/
sublancin 168	0.94	RiPP, 20,171	/	/
sporulation killing factor	1.00	RiPP, 22,954	/	/
alkylpyrone	0.51	PKS, 41,098	/	/

Note: The range of similarity scores for any cluster pairing is between 0 and 1, with 1 being a theoretical perfect score. / represents the most similar known cluster is not detected in genome. CDPS, tRNA-dependent cyclodipeptide synthases; NRPS, Non-ribosomal peptide synthetase; PKS, Polyketide synthase; RiPP, ribosomally synthesised and post-translationally modified peptide product; Other, Cluster containing a secondary metabolite-related protein that does not fit into any other category.

Table S3. The information of top ten largest peak areas identified by MS spectrometry .

**Peak ID**	**Name**	**RT (min)**	**Precursor *m/z***	**Height**	**Area**	**Adduct**
132	Bacillaene	9.373	563.3475	399405	2375143	[M-H20]+
141	Bacillaene	9.373	603.34025	172714	1082964	[M+Na]+
137	Bacillaene	9.373	581.3582	166754	1034800	[M+H]+
316	Angoletin	11.823	301.1412	67770	634216	[M+H]+
268	Gamabufotalin	9.747	425.2295	58077	525804	[M+Na]+
76	Scopoletin	6.382	193.04952	28307	360037	[M+H]+
312	Phthalic anhydride	11.679	149.02348	27394	285639	[M+H]+
182	Methyl hydroxyacetate	9.248	509.21456	49489	276038	[M+Na]+
71	2,6-Xylidine	5.562	122.09612	24291	238242	[M+H]+

257	Diferuloyl putrescine	9.553	441.20364	16911	171655	[M+H]+

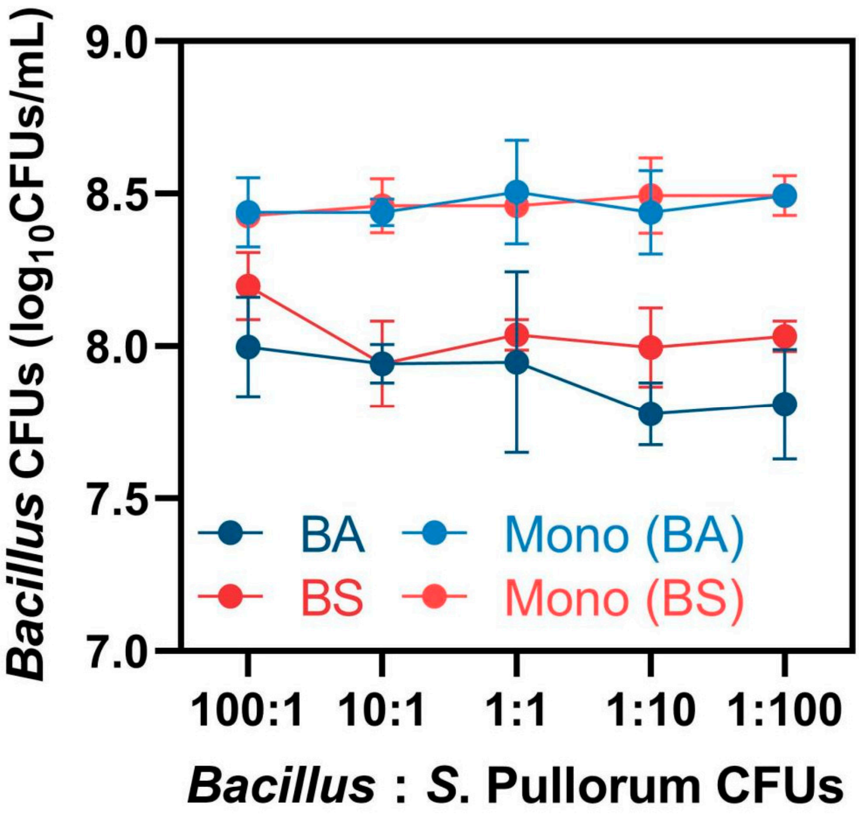

Figure S1. The inhibitory effect of *S*. Pullorum on the growth of both BA and BS was not significant difference across different initial cell numbers. The growth inhibition by *S*. Pullorum of BA and BS were not significant difference under different initial inocula. Student's t-test was performed to compare the means of cell counts between monoculture and co-culture, where statistically significant differences were determined ($*P < 0.05$). Data were presented as mean ± standard deviation (SD), n = 3.

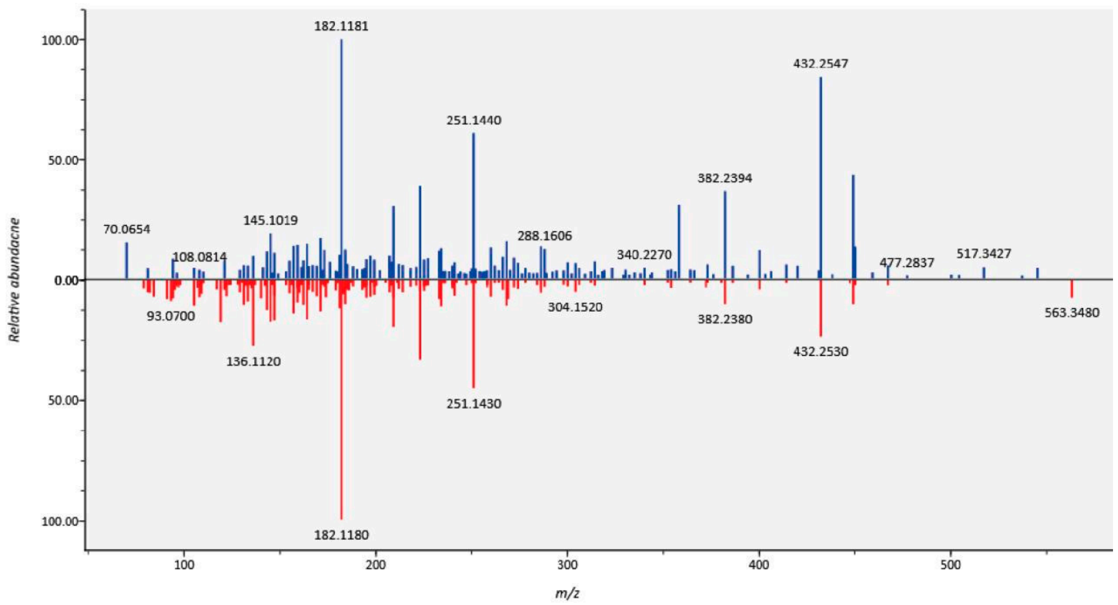

Figure S2. The comparison of purified antibacterial metabolite and bacillaene in MS/MS spectrometry. The blue bar represents the response value of purified antibacterial metabolite, and the red bar represents the response value of reference compound in the database. n = 6.

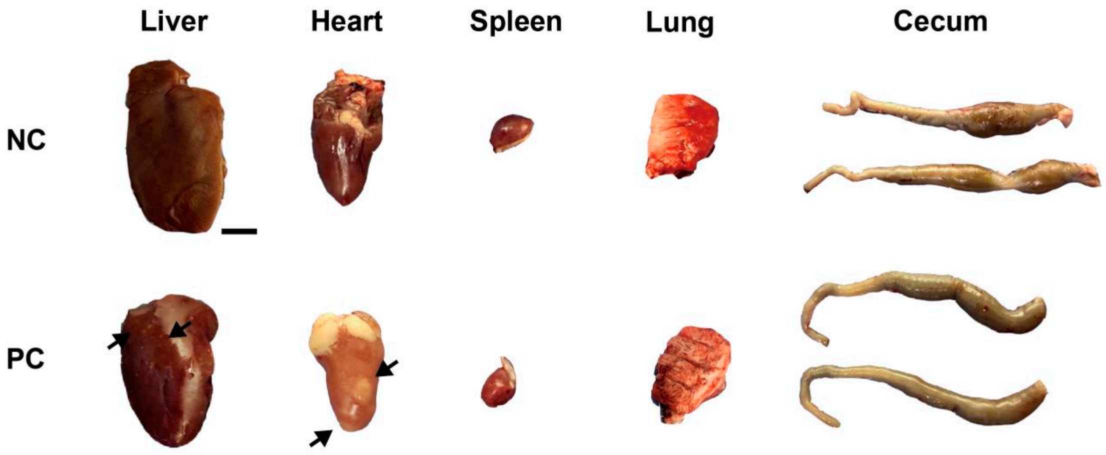

Figure S3. *S*. Pullorum infection led to the appearance of liver white spots and heart bulges. The pathological change area was pointed by arrow. Scale bar, 1 cm. NC, non-infected control. PC, positive-infected control.

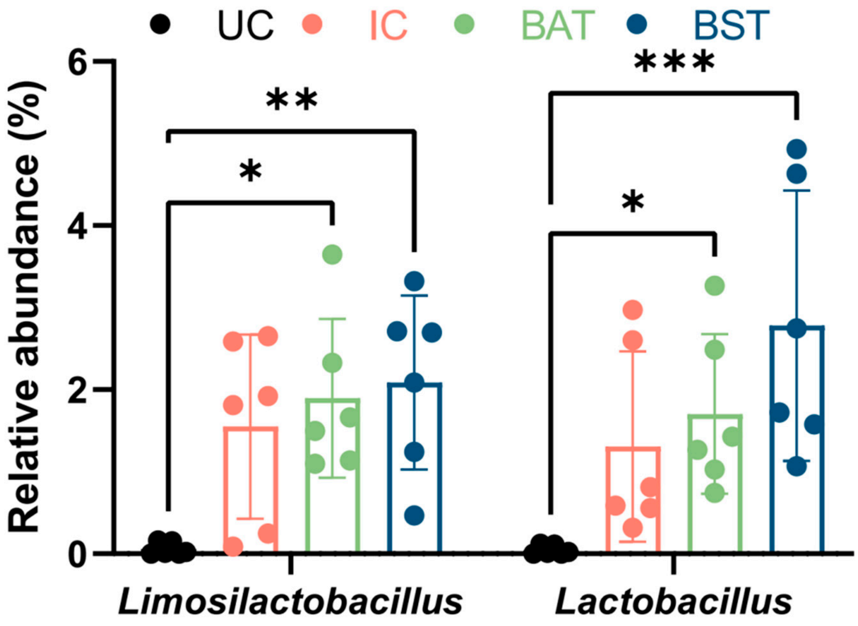

Figure S4. The relative abundance of *Lactobacillus* and *Limosilactobacillus* across different group. Two-way ANOVA was performed to compare the means of relative abundance between group, where statistically significant differences were determined (∗$P < 0.05$). Data were presented as mean ± standard deviation (SD), n = 6.

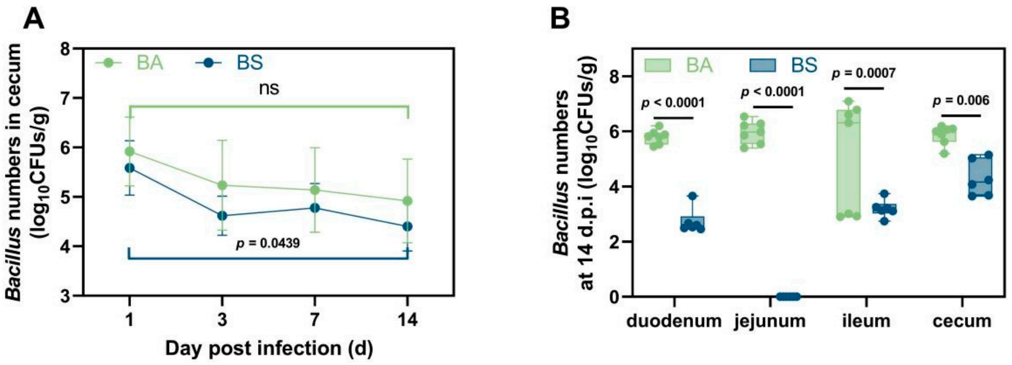

Figure S5. The temporal and spatial distributions of BA and BS in intestinal. (A) The colonization of BA and BS were stable in cecum within 14 days post infection. A comparison of Bacillus number was performed between 1 d.p.i and 14 d.p.i by unpaired student's t test. (B) BA showed stronger colonization capability to BS indifferent intestinal sections. Results represent the means ± SD. P values were calculated using ordinary two-way ANOVA with the LSD post hoc test.

Supplementary Methods

Genome Mining for Biosynthetic Gene Clusters in *B. subtilis* Using antiSMASH

The complete genome sequences of the *B. subtilis* strains were sequenced on an Illumina NovaSeq 6000 platform using a 2 ×150 bp read length and a sequencing depth of 400 ×. The sequenced data were filtered used by fastp v1.0 and assembled by SPAdes v4.2.0. The assembled sequences were subsequent used as direct input. The identification and annotation of biosynthetic gene clusters (BGCs) within the *B. subtilis* genomes were performed using antiSMASH version 7.0. The analysis was run with default parameters.
